# Long event-free survival after anti-BCMA CAR-T cell treatment for relapsed and refractory multiple myeloma patients: Two case reports

**DOI:** 10.1097/MD.0000000000025784

**Published:** 2021-05-07

**Authors:** Jinhuan Xu, Xi Ming, Chunyan Wang, Bi Xu, Yi Xiao

**Affiliations:** Department of Hematology, Tongji Hospital, Tongji Medical College, Huazhong University of Science and Technology, Wuhan, Hubei, P.R.China.

**Keywords:** B-cell maturation antigen, chimeric antigen receptor T cells, complete response, relapsed and refractory multiple myeloma

## Abstract

**Introduction:**

Chimeric antigen receptor T (CAR-T) cells targeting B-cell maturation antigen (BCMA) have been used in the treatment of relapsed and refractory multiple myeloma (RRMM). The response rate and the depth of responses induced by anti-BCMA CAR-T cells are impressive. However, despite this, remissions are not sustained, and the majority of patients eventually relapse.

**Patient concerns:**

Two patients with multiple myeloma (MM) were selected to enroll in a phase I study involving anti-BCMA CAR-T cells (ChiCTR-OPC-16009113) because they did not have the good effect after traditional treatment. One is a 48-year-old male patient who received a diagnosis of IgG lambda MM in June 2015, he has received 4 cycles of cyclophosphamide, bortezomib, and dexamethasone (CyBorD) and obtained a complete response (CR). Approximately 11 months later, the disease progressed. Subsequent treatment included regimens incorporating liposomal doxorubicin, bortezomib, and dexamethasone (3 cycles); the response was poor, and the disease kept progressing. Another 65-year-old female patient received a diagnosis of IgG lambda MM in September 2016, she has received induction therapy with 1 cycle of bortezomib and dexamethasone (VD) and 4 cycles of lenalidomide and dexamethasone, the response was poor.

**Diagnosis:**

Both patients were diagnosed with RRMM according to the International Myeloma Working Group criteria.

**Interventions:**

Both patients received infusions of anti-BCMA CAR-T cells following an induction chemotherapy regimen of cyclophosphamide and fludarabine.

**Outcomes:**

Both of them achieved a stringent CR at the 30th day with minimal residual disease-negative bone marrow by flow cytometry and serum monoclonal protein was undetectable at 4 and 10 months after cell transfusion. The CR has persisted in the 2 patients for >36 months.

**Conclusions:**

Our findings demonstrate the anti-BCMA CAR-T cell treatment is a feasible therapeutic option for patients with RRMM. Fewer early lines of treatment may be beneficial to maintain the efficacy of CAR-T cells.

**Trial registration:**

ChiCTR-OPC-16009113.

## 1 Introduction

Chimeric antigen receptor T (CAR-T) cell therapy targeting B-cell maturation antigen (BCMA) produced unprecedented results in heavily pretreated relapsed and/or refractory multiple myeloma (MM). Data on >300 patients with MM treated with anti-BCMA-directed CAR-T cells are available, and these numbers are rapidly increasing. Coming from different T-cell products used in different patients and settings, the response rates and depth of responses induced by anti-BCMA CAR-T cells are impressive. However, despite this, remissions are not sustained, and the majority of patients eventually relapse.^[1]^ So far, the longest median event-free survival (EFS) reported in the literature was 15 months.^[1,2]^ In this report, 2 patients with RRMM treated with infusions of anti-BCMA CAR-T cells had an ongoing stringent complete response (CR) for >36 months with minimal residual disease (MRD)-negative bone marrow as determined by flow cytometry, which may continue for a longer period of time. We analyzed the possible mechanisms for the continuous remission with combining the published literature.

## 2 Case presentation

### 2.1 Case 1

**Figure 1:**
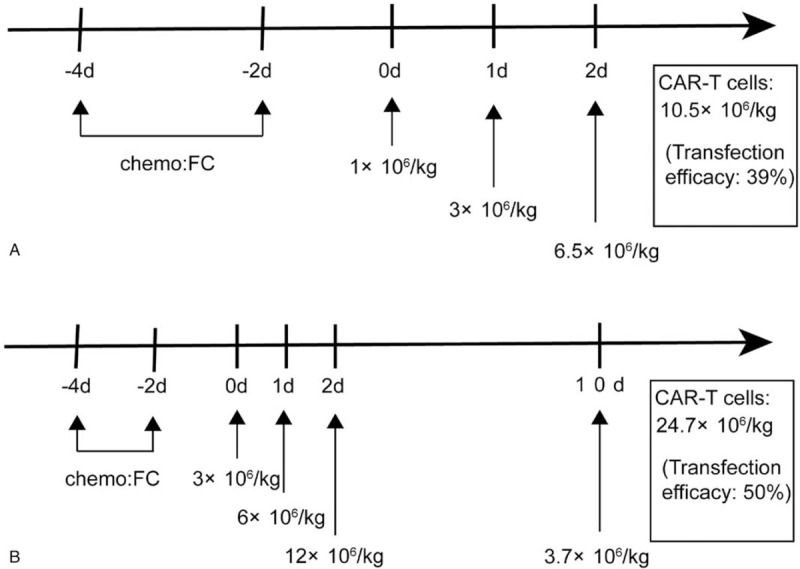
Protocol of anti-B-cell maturation antigen CAR-T cell infusions following chemotherapy. A protocol of CAR-T cell infusion in combination with chemotherapy. Chemotherapy included fludarabine and cyclophosphamide. CAR-T cells were infused at a total dose of 10.5 × 10^6^/kg for 3 days (A, Case 1) and 24.7 × 10^6^/kg for 4 days (B, Case 2). CAR-T = chimeric antigen receptor T.

**Figure 2:**
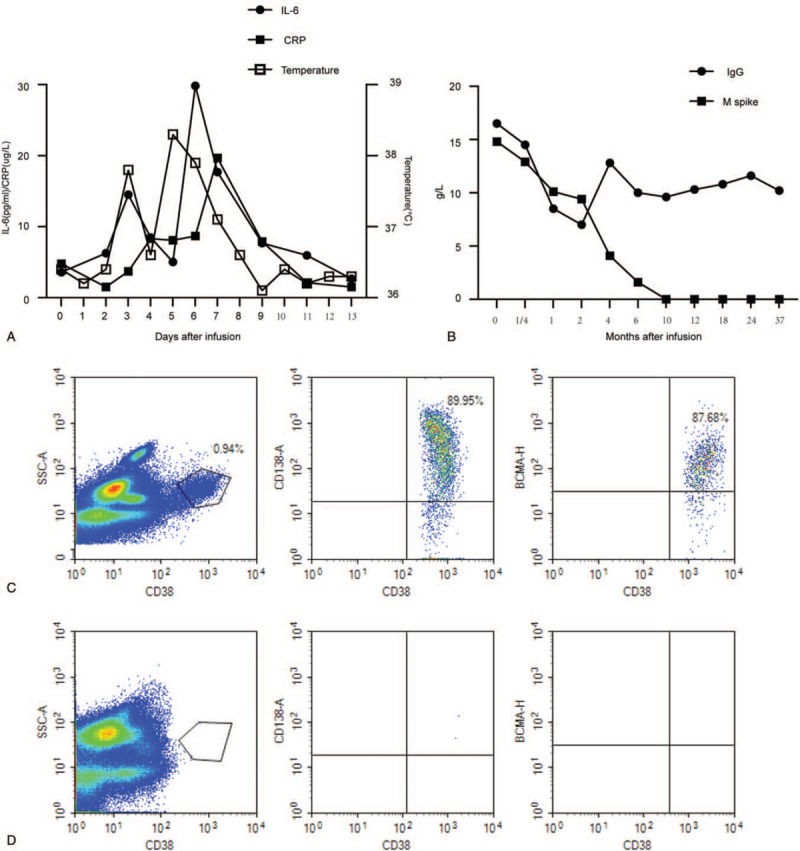
Measures of multiple myeloma burden and clinical responses to infusions of anti-BCMA chimeric antigen receptor T (CAR-T) cells of case 1. (A) A total of 72 hours after CAR-BCMA infusion, the patient became febrile. She was febrile for 4 days. The plot shows the maximum temperature for each day. The levels of IL-6 and CRP were elevated. (B) Panel B shows the trend in IgG and M spike concentrations after treatment with anti-BCMA CAR-T cell infusion. (C) Bone marrow cells were 0.94% plasma cells as shown by CD38 staining at 13 days before the anti-BCMA CAR-T cell infusion. BCMA expression per flow cytometry showed uniform BCMA expression on CD38-positive malignant plasma cells before the anti-BCMA CAR-T cell infusion. (D) No plasma cells were found among the bone marrow cells on day 30 after anti-BCMA CAR-T cell infusion. BCMA = B-cell maturation antigen, CRP = C-reactive protein, IL = interleukin, SSC-A = side scatter area.

A 48-year-old male patient received a diagnosis of stage IIIA (Durie-Salmon standard) IgG lambda MM in June 2015, after presenting with anemia and multiple bone lesions. He received induction therapy with 4 cycles of cyclophosphamide, bortezomib, and dexamethasone (CyBorD) and obtained a CR. However, he did not undergo further consolidation and maintenance treatment. Approximately 11 months later (September 2016), the disease relapsed, 3% of his bone marrow cells were plasma cells, and monoclonal protein was detectable in his serum and urine. Subsequent treatment included regimens incorporating liposomal doxorubicin, bortezomib, and dexamethasone (3 cycles). According to the International Myeloma Working Group response criteria,^[3]^ the response was poor, and the disease kept progressing. The patient refused to use other new drugs for treatment. In May 2017, after the failure of 2 previous lines of treatment, the patient was enrolled in a phase I clinical trial involving anti-BCMA CAR-T cell therapy. Before anti-BCMA CAR-T cell infusion, a bone marrow biopsy showed 3.5% myeloma involvement. CD38 and BCMA coexpression was revealed by flow cytometry (Fig. 2C). The treatment protocol and anti-BCMA CAR-T cell preparation (Supplemental Method) were the same as in our previous report.^[4]^ The patient was given fludarabine 25 mg/m^2^ and cyclophosphamide 20 mg/kg for 3 days (days −4 to −2) for lymphodepletion. Anti-BCMA CAR-T cells were infused on successive 3-day periods from day 0 (June 13, 2017), the effective anti-BCMA CAR-T cells totaled 10.5 × 10^6^/kg (Fig. 1A). One day after the completion of anti-BCMA CAR-T cell infusion, he became febrile. The patient was febrile for 4 days, with a maximum temperature of 38.3°C (Fig. 2a). The serum interleukin (IL)-6 and C-reactive protein (CRP) levels were elevated and peaked respectively on the 6th and 7th day postinfusion, which was also the time where toxicity culminated (Fig. 2A). Pancytopenia was observed but not central nervous system (CNS) toxicities. White blood cells and neutrophils returned to normal on the 9th day after infusion. No infusion of red blood cells or platelets was required during the entire treatment period. All coagulation and biochemistry parameters were normal over the whole treatment period. He experienced grade 1 cytokine-release syndrome (CRS). At the 7th day after anti-BCMA CAR-T cell infusion, we noted a striking decrease in serum monoclonal protein levels (Fig. 2B); 1 month later, multicolor flow cytometry did not detect MM cells for the plasma cell marker CD38 staining in the bone marrow (Fig. 2D), which confirmed a stringent CR. Normal plasma cells were also absent from the bone marrow 1 month after anti-BCMA CAR-T cell infusion. Serum monoclonal protein became undetectable 10 months postinfusion and remained undetectable until now (Fig. 2B). So far, the response to CAR-T cell infusion was still stringent CR at 37 months with MRD-negative bone marrow as determined by flow cytometry without any anti-myeloma treatment.

### 2.2 Case 2

**Figure 3:**
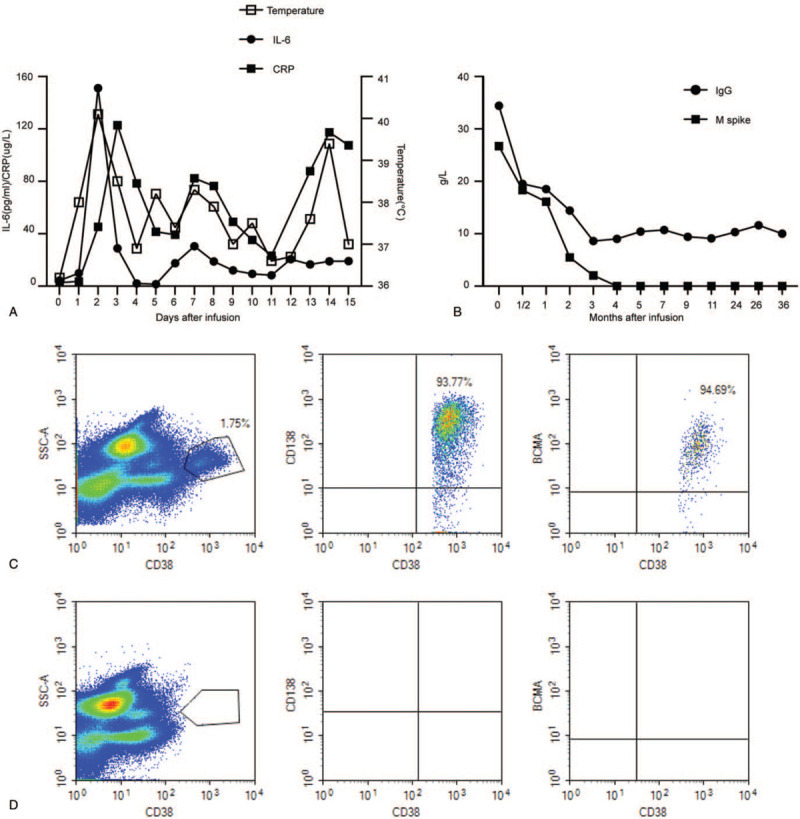
Measures of multiple myeloma burden and clinical responses to infusions of anti-BCMA chimeric antigen receptor T (CAR-T) cells of case 2. (A) A total of 4 hours after the second day of CAR-BCMA infusion, the patient became febrile. She was febrile for 13 days. The plot shows the maximum temperature for each day. The levels of IL-6 and CRP were elevated. (B) Panel B shows the trend in IgG and M spike concentrations after treatment with anti-BCMA CAR-T cell infusion. (C) Bone marrow cells contained 1.75% plasma cells as shown by CD38 staining at 10 days before the anti-BCMA CAR-T cell infusion. BCMA expression per flow cytometry showed uniform BCMA expression on CD38-positive malignant plasma cells before the anti-BCMA CAR-T cell infusion. (D) No plasma cells were found among the bone marrow cells on day 30 after anti-BCMA CAR-T cell infusion. BCMA = B-cell maturation antigen, CRP = C-reactive protein, IL = interleukin, SSC-A = side scatter area.

Another 65-year-old female patient received a diagnosis of stage IIA (Durie-Salmon standard) IgG lambda MM in September 2016, after presenting with anemia and multiple bone lesions. A bone marrow biopsy showed 36% myeloma involvement. Risk factors were noted, including P53 deletion, RB1 gene deletion, *MAF* gene deletion, and positive IgH/CCND1 fusion gene. She received induction therapy with 1 cycle of bortezomib and dexamethasone (VD). The patient developed severe diarrhea, fatigue, numbness of the limbs, and was unable to walk independently. After giving anti-diarrhea medication, correcting electrolyte disturbances and nutritional nerve treatment, diarrhea and fatigue improved significantly, but the numbness did not abate. There was a high possibility of nerve damage caused by bortezomib, and subsequent treatment included regimens incorporating lenalidomide and dexamethasone (TD 4 cycles). Although the numbness improved significantly, according to International Myeloma Working Group response criteria,^[3]^ the patient's disease remained stable. In August 2017, the patient was enrolled in the aforementioned clinical trial and received anti-BCMA CAR-T cell treatment. Before anti-BCMA CAR-T cell infusion, a bone marrow biopsy showed 6% myeloma involvement, and CD138 and BCMA coexpression was revealed by flow cytometry (Fig. 3C). The treatment protocol was the same as that in patient 1; the effective cells totaled 24.7 × 10^6^/kg (Fig. 1B). Six hours after the second infusion, she developed fever (38°C, peaked at 40.1°C on the next day), and lasted for 14 days) that was associated with elevated serum IL-6 and CRP levels. The peak of serum IL-6 (35 times higher than baseline) was detected on the second-day postinfusion, which was just the time where toxicity culminated (Fig. 3A). After the third reinfusion, this patient also had grade 1 delirium without other neurologic toxicities, which was treated with dexamethasone, diazepam, and mannitol. Neurological symptoms were relieved, and there was no long-term neurological dysfunction. Pancytopenia was observed, and the white blood cells and neutrophils returned to normal 11 days after the infusion. All coagulation and biochemistry parameters were normal over the whole treatment period. She experienced grade 1 cytokine-release syndrome and grade 1 central nervous system toxicities. Bone marrow MM cells were not detected by multicolor flow cytometry with CD138 staining 30 days after CAR-T cell infusion (Fig. 3D). Serum monoclonal protein became undetectable at 4 months postinfusion and remained undetectable until now (Fig. 3B). She also had an ongoing stringent CR at 36 months with MRD-negative bone marrow, as determined by flow cytometry, without any anti-myeloma treatment.

## 3 Discussion

The 2 patients with RRMM achieved stringent CR after the infusion of anti-BCMA CAR-T cells, with an EFS duration of >36 months, without any anti-myeloma treatment, that is likely to continue. So far, the longest median progression-free survival reported in the literature was 15 months.^[1,2]^ Anti-BCMA CAR-T cell therapies have shown impressive anti-myeloma activities, but relapse is still inevitable in most patients. The underlying mechanisms of relapse in patients with MM treated with anti-BCMA CAR-T cells may be due to T cell-related, tumor-related, and microenvironmental factors.^[1]^ The treatment efficacy was found to mainly correlate with the dose of CAR^+^ T cells infused and the degree of in vivo expansion after CAR-T cells therapy in MM.^[5–7]^

Our patients achieved longer EFS, which may be related to several factors. First, they had fewer early lines of treatment, causing less damage to T cell function. Published data have shown that the number of median prior lines of treatment in all RRMM patients exceeded 3 before they were treated with anti-BCMA CAR-T cells,^[1]^ whereas our 2 patients received only 2 previous lines of treatment. Research has shown that cancer and its treatment can hamper T cell fitness,^[7]^ and starting the manufacturing process from fitter T cells may prolong in vivo CAR-T cell persistence, possibly limiting BCMA^+^ relapses.^[1]^ In addition, in patients with MM who receive fewer early treatments, the number of early memory T cells may be higher.^[8]^ There is initial evidence that there is a preferential transduction of the CAR in early memory cells (eg, P-Bcma-101).^[9]^ In all current clinical studies of anti-BCMA CAR-T cell therapy for RRMM, the one with the fewest median previous treatment lines obtained the highest CR rate (74%) and the longest median EFS (15 months).^[1,2]^ Second, lymphodepleted chemotherapy was more intense, although lymphodepletion was not necessary to obtain responses and CAR-T expansion. Lymphodepleted patients showed better CAR-T cell kinetics and outcomes.^[6]^ The lack of competition by other lymphocytes allows CAR-T cell expansion and persistence.^[10]^ In a recent report, a higher intensity of fludarabine-cyclophosphamide lymphodepletion correlated with the progression-free survival of non-Hodgkin lymphoma patients treated with anti-CD19 CAR-T cells.^[11]^ Shi et al^[12]^ investigated that more intense lymphodepletion protocols may support better CAR-T cell expansion. The combination of cyclophosphamide and fludarabine was used to deplete lymphocytes in our study, the dosage was higher than that in other studies, and the peripheral lymphocyte count of the 2 patients was very low (0.11 × 10^9^ cells/L and 0.16 × 10^9^ cells/L, respectively) before infusion, which provides better conditions for the proliferation of anti-BCMA CAR-T cells. Third, compared with other patients, larger doses of anti-BCMA CAR-T cells were infused into the 2 patients. According to reports in the literature, multiple studies have shown that the amount of cells infused was positively correlated with the therapeutic effect.^[5,6]^ Finally, the low tumor burden of the 2 patients may be beneficial to their outcomes, although no studies have shown that the treatment efficacy of anti-BCMA CAR-T cells was related to MM tumor burden. Another study has shown that the treatment efficacy of CAR19/22 T cell cocktail therapy in patients with refractory/relapsed B cell malignancies was found to correlate with tumor burden.^[13]^ These are speculations combined with findings from the literature, and the exact mechanism needs further basic and clinical research.

At present, most patients have undergone at least 3 lines of previous treatment before anti-BCMA CAR-T cells treatment, with a maximum of 29 lines.^[1]^ Heavily pretreated patients are usually multidrug refractory and show aggressive patterns of relapse. In this context, clinical deterioration may preclude the actual CAR-T cell infusion.^[6]^ Ongoing trials are looking at the efficacy of CAR-T cell therapy in earlier lines of treatment,^[1]^ which is given as second-line treatment in high-risk patients who have had either early relapse or suboptimal responses to first-line therapy. CAR-T cell therapy allows some patients treatment-free intervals, when they often enjoy a high quality of life without therapy-related toxicities. We look forward to more patients with RRMM, like our 2 patients, achieving longer EFS with CAR-T cell treatment.

## Acknowledgments

The authors thank all members of the study team, the patient, and their family, and Wuhan Bio-Raid Biotechnology Co., LTD.

## Author contributions

J.X analyzed the data and wrote the manuscript. XM and BX Took care of the patient, C.W conducted the flow cytometry. YX revised the manuscript and was in charge of the final approval of the manuscript. J.X, XM and YX performed the experiments. YX conceived and designed the study. All authors read and approved the final manuscript.

**Conceptualization:** Jinhuan Xu, Yi Xiao.

**Data curation:** Jinhuan Xu, Xi Ming, Chunyan Wang.

**Formal analysis:** Xi Ming.

**Funding acquisition:** Jinhuan Xu, Yi Xiao.

**Investigation:** Xi Ming, Chunyan Wang.

**Project administration:** Jinhuan Xu, Chunyan Wang, Bi Xu, Yi Xiao.

**Supervision:** Bi Xu.

**Visualization:** Xi Ming, Bi Xu.

**Writing – review & editing:** Jinhuan Xu.

## Supplementary Material

Supplemental Digital Content
