# Supplementary material for: Long event-free survival after anti-BCMA CAR-T cell treatment for relapsed and refractory multiple myeloma patients: Two case reports
Source: Medicine (Baltimore). 2021 May 7;100(18):e25784. doi: 10.1097/MD.0000000000025784 (PMC8104258; doi:10.1097/MD.0000000000025784)
Supplement: Supplemental Digital Content [file medi-100-e25784-s001.docx]

**CAR T-cell production**

Autologous peripheral blood mononuclear cells (PBMCs) were cultured with an anti-CD3 monoclonal antibody to induce T-cell proliferation. The anti-BCMA CAR was encoded by a lentiviral vector containing a murine anti-BCMA single-chain variable fragment, a CD8a hinge, the CD28 transmembrane regions and intracellular domain, and CD3- ζ T-cell activation domain(**Figure 1.**). the anti-BCMA CAR-T cells were cultured for 14 days before infusion.

**Figure 1.**


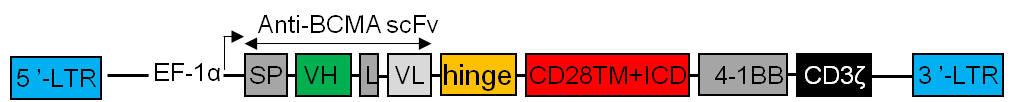


**Figure 1. Construction of BCMA-specific CAR and detecting their expression in CAR-transduced T cells.**

A. Schematic diagram of anti-BCMA CAR vector. SP: signal peptide, VH: variable H chain, L: linker, VL: variable L chain.
